# The effects of the depth of fertilization on losses of nitrogen and phosphorus and soil fertility in the red paddy soil of China

**DOI:** 10.7717/peerj.11347

**Published:** 2021-05-18

**Authors:** Kun Hou, Ying Huang, Xiangmin Rong, Jianwei Peng, Chang Tian, Yongliang Han

**Affiliations:** College of Resources and Environmental, Hunan Agricultural University, Changsha, China

**Keywords:** Deep fertilization, Nitrogen, Phosphorus, Losses

## Abstract

Nitrogen (N) and phosphorus (P) losses from agroecosystems are dominant nonpoint pollution. To minimize the losses of N and P, the optimal depth of fertilization was explored using a soil column study with the red paddy soil as the research objects. The losses of N and P were measured under five depths of fertilization (0, 5, 7.5, 10, and 12.5 cm) as well as no fertilization. The results showed that ammonia volatilization was significantly decreased with increasing fertilization depth within 010 cm, and there was no significant difference among the 10 cm, 12.5 cm, and no-fertilization treatments. Comparing with surface fertilization (0 cm), N and P losses by runoff could be reduced by 30.767.1% and 96.998.7% respectively by fertilization at 512.5 cm. In addition, deep fertilization (512.5 cm) did not increase N and P losses by leaching at the depth of 40 cm. Total N and P contents in the tillage layer of soil were increased by 5.1 to 22.8% and by 1.0 to 7.5%, respectively. Fertilization at 10cm depth has the potential to minimal environmental impact in the red paddy soil of south China, at this depth, NH_3_ volatilization was reduced by 95.1%, and N and P losses by runoff were reduced by 62.0% and 98.4%, respectively, compared with surface fertilization.

## Introduction

The application of fertilizers must meet the demands of crop growth, help achieve greater yields, and contribute to food required by an increasing population (Spiertz, 2010; Ke et al., 2017). In China, improvements of grain yield have mainly relied on the application of chemical fertilizers, which often exceed nitrogen (N) inputs on the farmland, that is, approximately 300kg/hm^2^ for rice monoculture, and over 350kg/hm^2^ for paddy rice in the Taihu Lake region (Qiao et al., 2013). China has been the largest N fertilizer consumer, with 30% of the artificial N-fixation in the world applied on only 7% of the world land base. The N and P losses from the agroecosystem have resulted in serious damage to the environment, such as acid rain, water eutrophication, and loss of biodiversity (Hua et al., 2017; Zhao et al., 2012; Humbert et al., 2016; Zeng et al., 2016; Cissa et al., 2019). Rice was identified as the critical source of nutrient losses to water. The N losses from rice systems was several fold greater than from wheat or maize systems (Linquist et al., 2012). Control of the primary loss pathways of N and P and a reduction of the damage to the environment are very critical factors for rice production. Nitrogen management in the rice production regions of China was one of the national and global interests (Wu et al., 2015).

Ammonia volatilization has been identified as one of the main pathways of N loss, accounts for 1060% of the applied N, and the amount of NH_3_ volatilization was affected by the N form, temperature, soil pH, and plant residues (Soares, Cantarella & Menegale, 2012; Liang et al., 2007). In the major rice cultivation areas of China, NH_3_ volatilization dominates the amount of N loss, which accounted for 42.272.0% of the applied N. The second pathway of N loss is runoff, which was 22.238.4% of the applied N (Zhang, Liu & Hou, 2019). Previous studies have identified that the runoff-N was the main source of N pollution in water bodies, which occupied as high as 57% of N (Norse & Ju, 2015; Gu et al., 2013). Leaching, another pathway for N loss, results as N seeps through soil into local water systems and accounted for 5.822.7% of the applied N (Zhang, Liu & Hou, 2019). Unlike N, which with strong mobility, P is with weak mobility due to the quick adsorption by soil materials (Fe^3+^, Al^3+^, Ca^2+^ and so on), there are few P loss by leaching, and the majority of P-loss was transferred to runoff by water movement (Hua et al., 2017; Liu et al., 2016). The runoff-P from agroecosystems has become a primary source of P in surface water, estimated at over 60% of P in surface water (Norse & Ju, 2015; Hua et al., 2017).

Present work on controlling sources of agricultural non-point pollution mainly focus on fertilizer reduction, interception, and end-of-pipe treatments (Yang et al., 2013), but the method of fertilizer delivery also warrants study. Urease inhibitors, coated urea, bio-charcoal, ecological ditches, and constructed wetlands are among the approaches studied. It was clear that these measures reduce environment pollution significantly, but at an increased cost of production for famers. In the major rice cultivation areas of south China, the combination of high-intensity production, surplus N and P, surface fertilization, heavy rain, and well-developed water systems leads to serious non-point source pollution of N and P. There is also an urgent need to establish a simple, low-cost and environmental friendly production method for rice as the labor shortage becomes more and more serious in these areas. At present, the machine of transplanting rice seedlings with synchronous fertilization liberated the labor force of famers, and made the process of deep fertilization simple and easy to practice (Zhong et al., 2019). We hypothesized that deep fertilization inhibited N and P loss, but these the impact of different fertilization depths on N and P losses has not yet been determined.

In order to minimize the losses of N and P, decreased the environmental impact caused by fertilization, the optimal depth of fertilization was explored using a soil column study, which was designed to investigate the effect of different fertilization depth on N and P losses. The typical paddy soil in this area was chosen to be the research object, and fertilization depths of 0, 5, 7.5, 10 and 12.5cm were selected. Pathways of NH_3_ volatilization, N and P loss vs storage in the surface and leaching water, and distribution of N and P in the different soil layers of the soil column were measured in this trail. The study investigated N and P losses under different depths of fertilization as a potential means to reduce agricultural non-point source pollution of N and P.

## Materials & Methods

### Experimental materials and design

**Figure 1:**
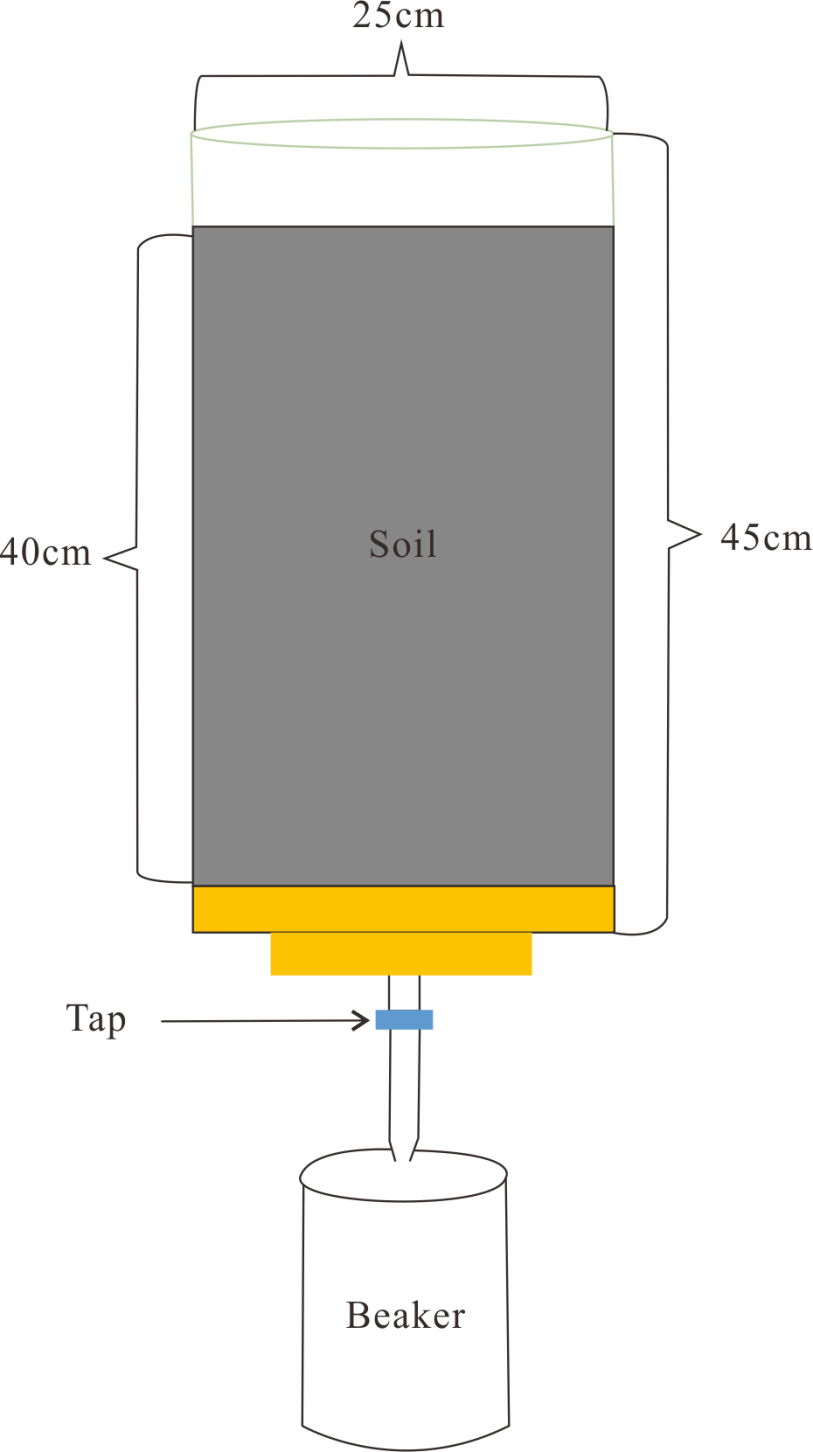
Soil column installation.

A soil column study was used to test the effect of different fertilization depths on N and P loss. Red paddy soil was dug from the field and placed in a PVC cylindrical containers of 45cm height and 25cm diameter. The height of the soil column was 40cm. The bottom of each cylindrical container was closed, and a tap was installed to collect leeching water (Fig. 1). The red paddy soil, one of the typical paddy soils in the rice cultivation areas of south China, had the following agrochemical characteristics: soil pH of 4.9, total N content of 0.66 g/kg, total P content of 0.39 g/kg, and total potassium content of 10.2 g/kg. Each fertilization treatment, described below, was replicated three times.

The fertilizer was a three-nutrient compound fertilizer (NP_2_O_5_K_2_O, 27.17.418.5%) used for rice cultivation in south China. The amounts of fertilizer used were as below: N 0.18, g/kg soil; P_2_O_5_, 0.05 g/kg soil; and K_2_O, 0.12 g/kg soil. The fertilizer was applied at one of five depths: 0, 5, 7.5, 10, and 12.5 cm. These five depths of fertilization constitute the five treatments in this trial; there was also a sixth treatment in which no fertilizer was applied. After fertilization, distilled water was slowly added to the soil column until the water infiltrated into the bottom of the soil column and there was two cm of standing water that remained on the soil surface. After 4h, the surface water and leachate were collected and the N and P concentrations determined. Every 3 days, this process was repeated until there was no significant difference among the fertilization depth treatments. The rate of NH_3_ volatilization was measured daily after fertilization until there was no significant difference among treatments. At the end of the trial, soil layers of 020, 2030, and 3040 cm were collected and the total N and P contents of each layer determined.

### Sampling and analysis

Soil column NH_3_ fluxes were measured twice each day (9:00 to 11:00 and 15:00 to 17:00) using the continuous air-flow enclosure method described in detail by Yong Xie and others (2019). The determination of NH_3_ fluxes was conducted for 20 days. Total NH_3_ volatilization was calculated by NH_3_ fluxes multiplied the surface area of soil column.

The surface water was collected by syringe, and the leachate was collected by plastic bottle. The concentration of total N was determined using alkaline potassium persulfate oxidation under ultraviolet spectrophotometry (Wei, 2002). The collected water sample (10 mL) was transferred to a 50-mL test tube, diluted to 25 mL with distilled water, and the alkaline potassium persulfate solution (5 mL) was added; the sample was then transferred to a high-temperature steam sterilizer and held for 30 min at 120C. When the mixture had cooled to room temperature, one mL of HCL (1.2 mol/L) was added, and the same brought to constant volume (50 mL) with the addition of distilled water. Samples were processed at 220 nm and 275 nm. The total N concentration was calculated according to a standard curve.

The concentration of total P was measured using potassium persulfate oxidation molybdenum blue colorimetric method (Wei, 2002). Water samples of 40 mL were transferred to a 50-mL test tube, and four mL of potassium persulfate solution (5%) was added. Then the mix was transferred to high-temperature steam sterilizer and held for 30 min at 120C. When the mixture had cooled down to room temperature, one mL of ascorbic acid (100 g/L) was added, then two mL of molybdenum antimony solution was added, and the sample brought to constant volume (50 mL) with the addition of distilled water. Colorimetry was measured at 700 nm 15 min later. The total P concentration was calculated according to a standard curve.

Soil samples were collected from the columns at layers of 020, 2030, and 3040 cm depth. Total N of soil samples was measured using Kjedahl digestion-distillation. The total P of soil samples was measured using the colorimetric molybdate-ascorbic acid method after the soil samples were digested with H_2_SO_4_-HClO_4_.

### Statistical analyses

All statistical analyses were performed using the SPSS software (Statistical Product and Service Solutions V13.0, USA). One-way analysis of variance was performed to assess the significance of differences in parameters among different treatments using Duncans new multiple range test. Different letters in the figures and tables indicate statistically significant differences at *P*<0.05.

## Results

### NH_**3**_ volatilization in different fertilization depths

**Figure 2:**
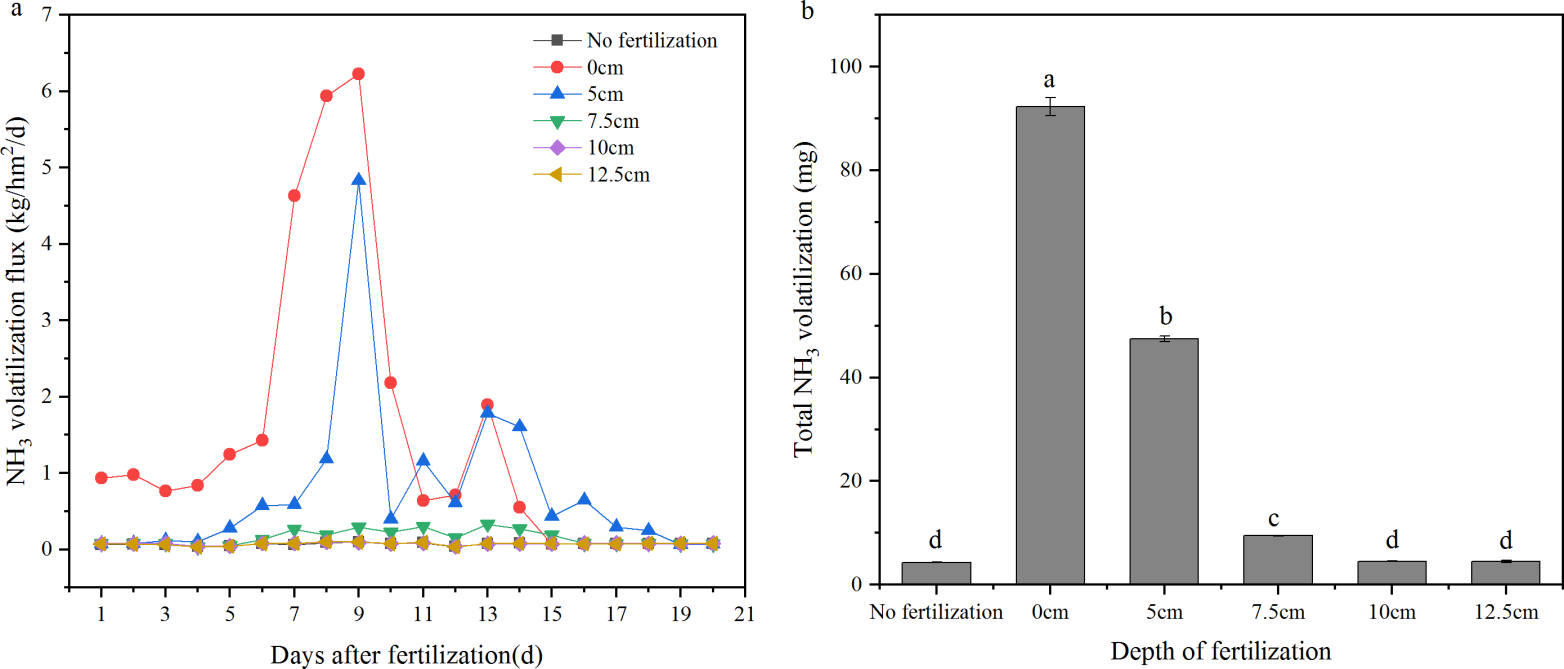
NH_3_ volatilization of the treatments with different depth of fertilization. (A) NH_3_ volatilization flux (kg/hm^2^/d). (B) Total NH_3_ volatilization (mg). Different letters indicate significant differences at *P*<0.05 among the treatments with different depth of fertilization.

The NH_3_ volatilization flux was decreased with increased depth of fertilizer application within 010 cm, and there was no significant difference between 10 cm and 12.5 cm (Fig.2). For the 0 cm and five cm depths of fertilization, NH_3_ volatilization flux was relatively stable within the first 4 days, it showed a raising trend from the 5th day, and reached to the peaks in the 9th day, afterwards, NH_3_ volatilization flux decreased rapidly in the 10th day. The NH_3_ volatilization flux showed a small peak in the 13th day, which may be due to the high temperature of the environment. afterwards, The NH_3_ volatilization flux were decreased. The NH_3_ volatilization of the 7.5 cm treatment was higher than 10 cm and 12.5 cm treatments, but showed no peak during the test period. The total NH_3_ volatilization was calculated, and the results showed that surface application of fertilizer (0 cm) had the largest loss of N by NH_3_ volatilization compared with the other treatments. The NH_3_ volatilization rate was significantly inhibited by deep fertilization. There were significant differences among the 0, 5, 7.5, and 10 cm treatments, and there was no significant difference among the treatments of 10 cm, 12.5 cm, and no fertilization. The NH_3_ volatilization rate in the 5, 7.5, 10, and 12.5 cm treatments was reduced by 48.5, 89.8, 95.1, and 95.1%, respectively, compared with the 0 cm treatment (Fig. 2; Table 1).

### N concentration in the surface and leaching water of different fertilization depths

**Figure 3:**
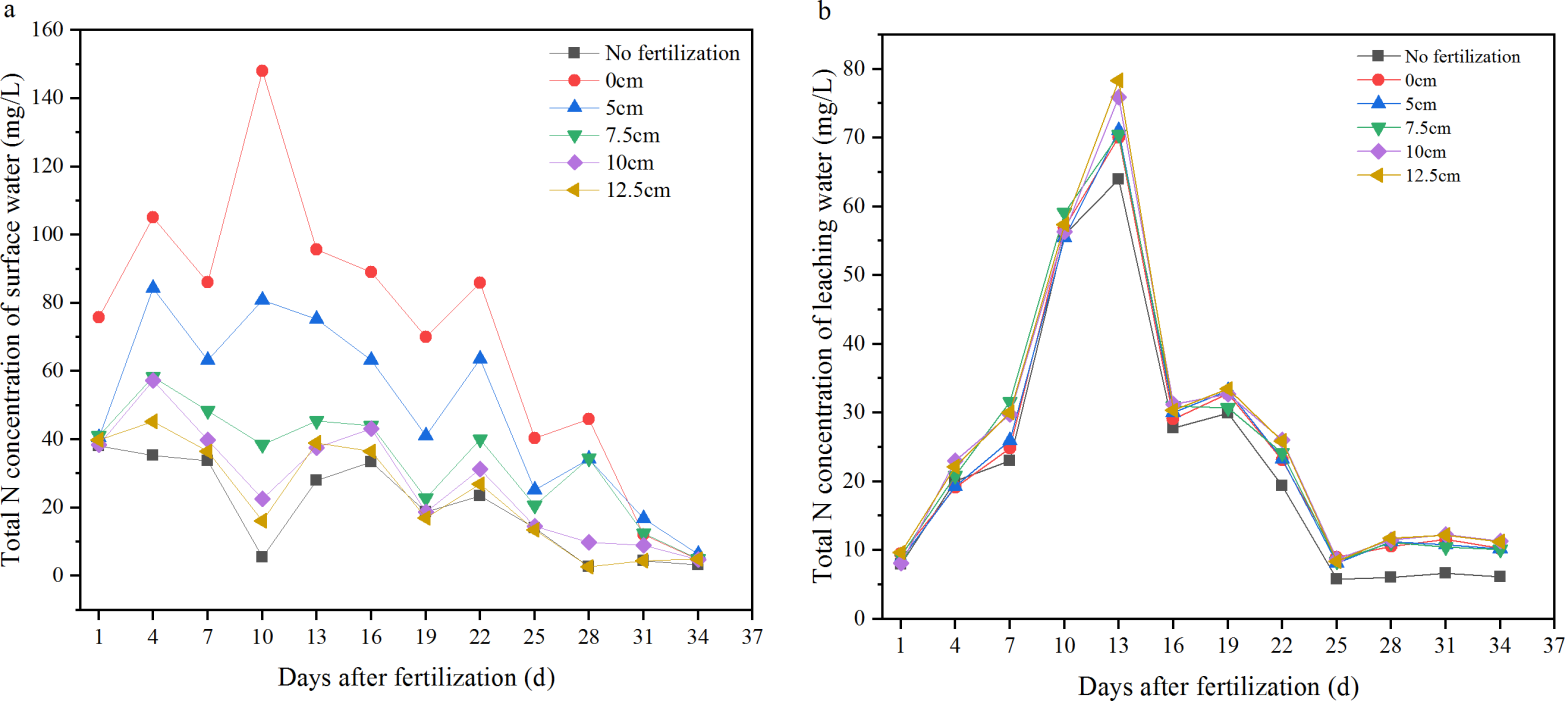
Variations of total N concentration in the surface and leaching water. (A) Surface water; (B) leaching water.

**Figure 4:**
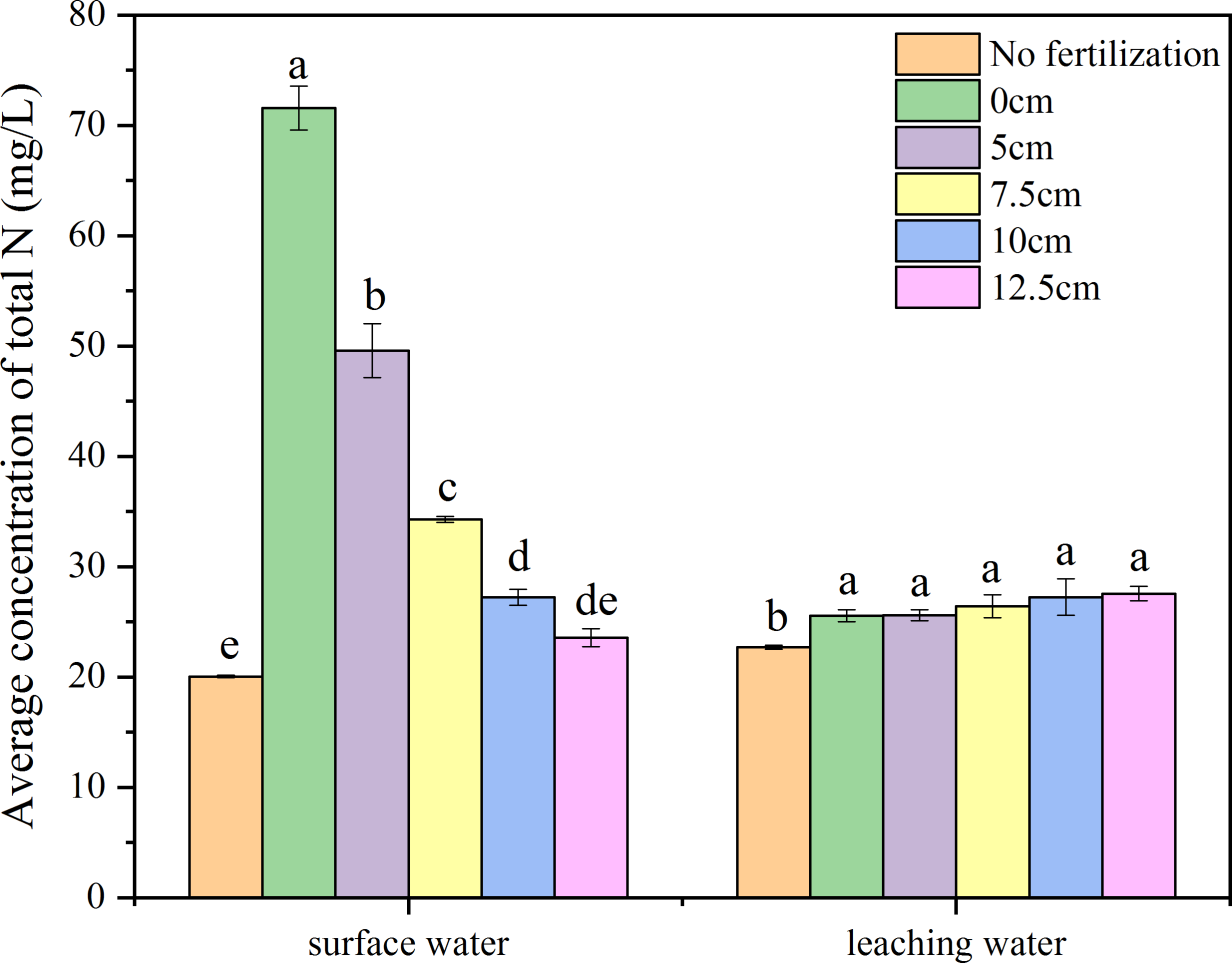
Average concentration of total N in the surface and leaching water. Different letters indicate significant differences at *P*<0.05 among the treatments with different depth of fertilization.

The N concentrations in the surface water increased at first and then gradually decreased after fertilizer application (Fig. 3A). In the first day after fertilization, there was no significant difference among the 0, 5, 7.5, 10, and 12.5 cm fertilization treatments; from the 4th day, the deeper the fertilization depth, there were lower N concentrations in the surface water. This lasted until the 34th day, on which there were no significant differences in the N concentration of surface water among all treatments. The results showed that, with greater depth of fertilization, the average N concentration in surface water decreased significantly (Fig. 4). There were significant differences among the 0, 5, 7.5, and 10 cm treatments, though there was no significant difference between 10 and 12.5 cm, or between 0 and 12.5 cm. The N concentration in the surface water after fertilizer applied at 10 cm was significantly higher than that of no fertilization. The runoff-N losses could be reduced by 30.767.1% through deep fertilization (512.5 cm) compared with surface fertilization (Table 1).

**Table 1:** Effects of deep fertilization on N, P losses and tillage layer fertility compared with surface fertilization.

	0 cm	5 cm	7.5 cm	10 cm	12.5 cm
NH_3_ volatilization reduced by		48.5%	89.8%	95.1%	95.1%
runoff-N losses decreased by		30.7%	52.1%	62.0%	67.1%
leaching-N losses decreased by		0.2%	3.4%	6.6%	7.9%
runoff-P losses decreased by		96.9%	98.2%	98.4%	98.7%
leaching-P losses decreased by		6.3%	7.4%	6.5%	8.3%
Total N in tillage layer increased by		22.8%	25.0%	23.9%	5.1%
Total P in tillage layer increased by		7.5%	4.2%	2.0%	1.0%

After the fertilizer application, the N concentrations in the leaching water of all treatments were increased until the 13th day. The N concentrations in the leachate then all decreased until the 25th day. The N concentrations in the leaching water were relatively stable within the 25th to 34th day. There was no significant difference in the N concentration in leachate among the 0, 5, 7.5, 10, and 12.5 cm treatments; the N concentrations in the leachate of all fertilization treatments (012.5 cm) were significantly higher than that of no fertilization (Figs. 3B, 4).

### P concentration in the surface and leaching water of different fertilization depths

**Figure 5:**
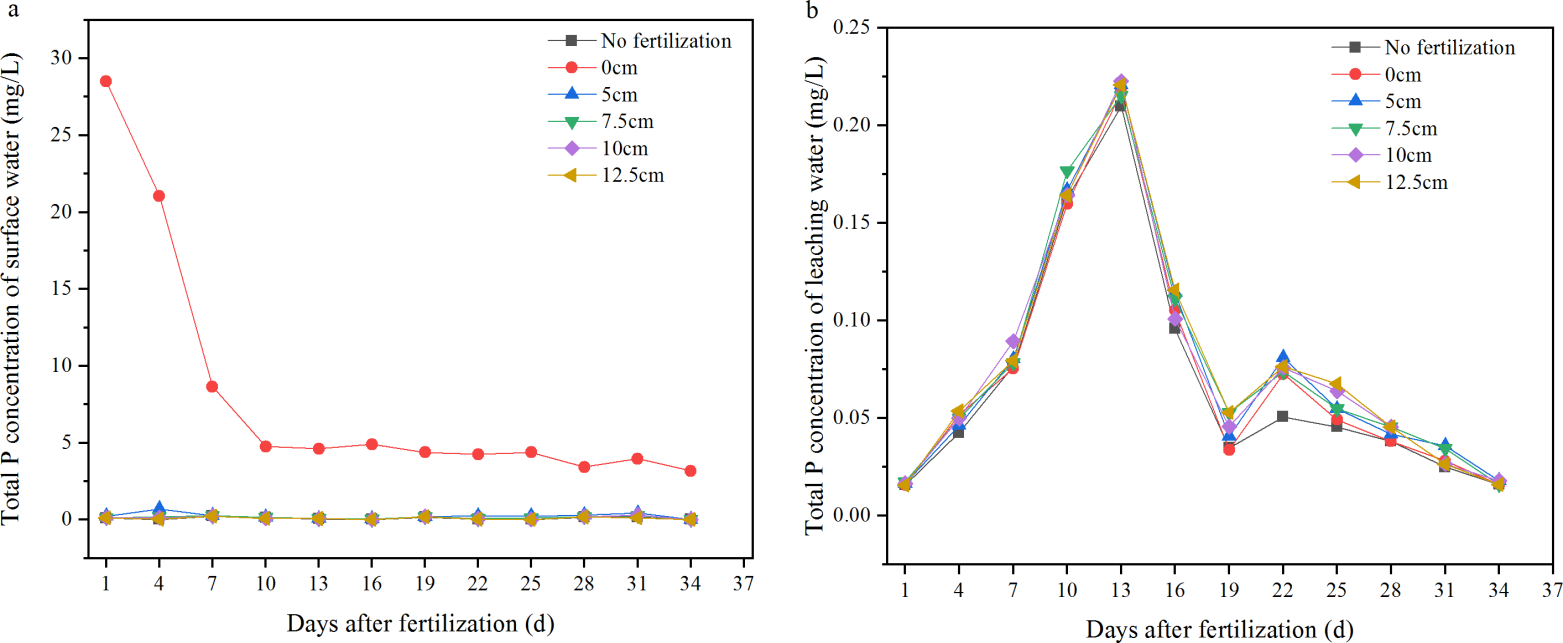
Variations of total P concentration in the surface and leaching water. (A) Surface water; (B) leaching water.

**Figure 6:**
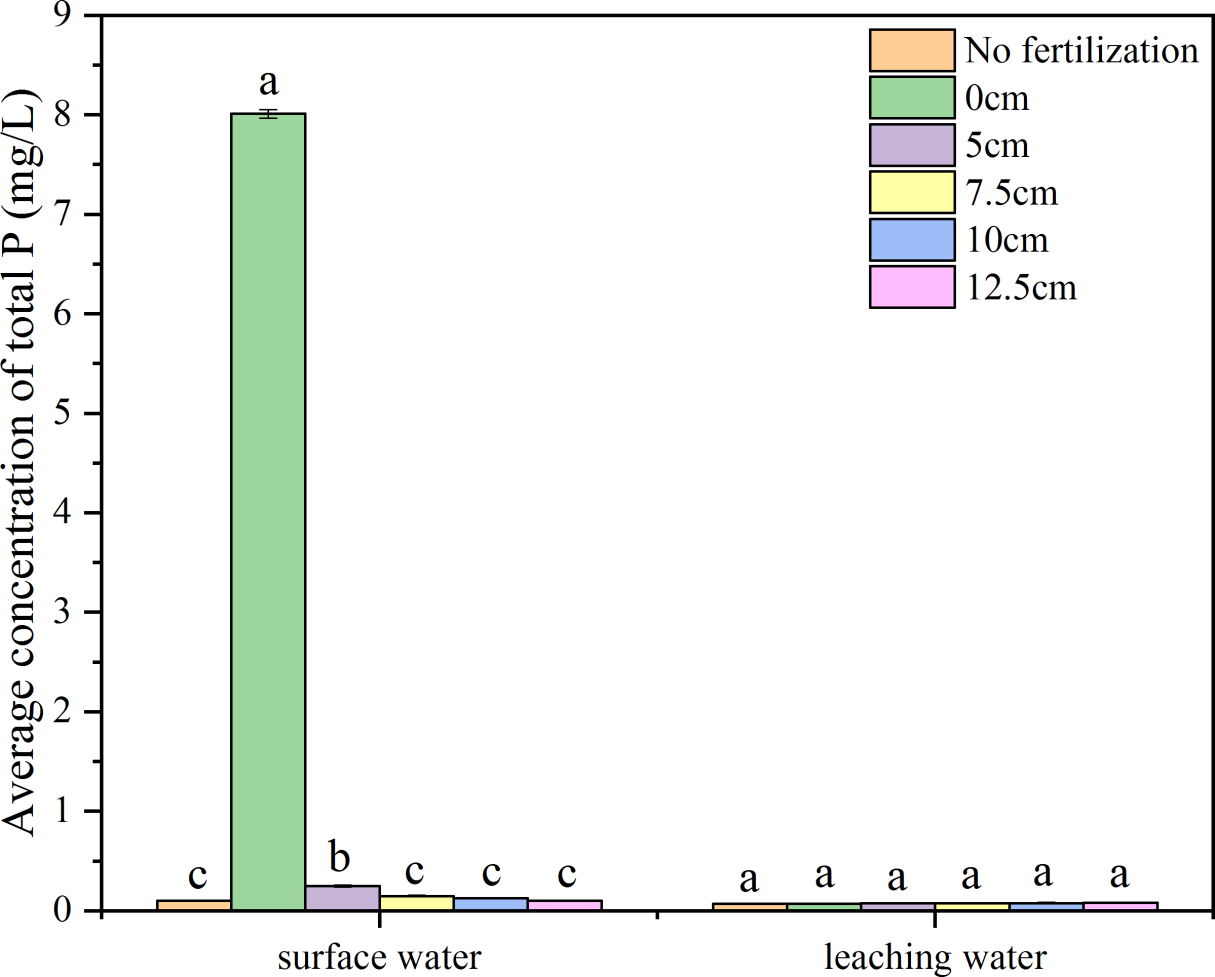
Average concentration of total P in the surface and leaching water. Different letters indicate significant differences at *P*<0.05 among the treatments with different depth of fertilization.

The P concentration in the surface water was significantly decreased by deep fertilization (512.5 cm) compared with surface fertilizer application (Figs. 5A, 6). The first day of fertilization showed the highest P concentration, reaching 28.5 mg/L in the surface water of 0 cm fertilization; P concentration then decreased to 4.2 mg/L on the 10th day and lasted to the end of the trial. The P concentrations in the surface water of deep fertilization treatments were far less than that of surface fertilization, and the variations were much less than that of surface fertilization as well. There was a significant difference in the average P concentration in surface water between the 0 cm and other treatments, and there was significant difference between the five cm and each of the 7.5 cm, 10 cm, 12.5 cm, and no fertilization treatments. There was no significant difference among 7.5 cm, 10 cm, 12.5 cm, and no-fertilization treatment (Fig. 6). The runoff-P losses could be reduced by 96.998.7% through deep fertilization (512.5 cm) compared with surface fertilization (Table 1).

The P concentrations in the leaching water were raised to about 0.22 mg/L in the 13th day after the fertilizer application, with an initial P concentration of approximately 0.02 mg/L. Phosphorus concentrations then decreased to 0.02 mg/L in the 34th day. There was no significant difference among the 0 cm, five cm, 7.5 cm, 10 cm, 12.5 cm, and no-fertilization treatments to indicate that there was no significant increase in the leaching-P losses under deep fertilization.

### Total N and P content in the different soil layers of different fertilization depths

**Figure 7:**
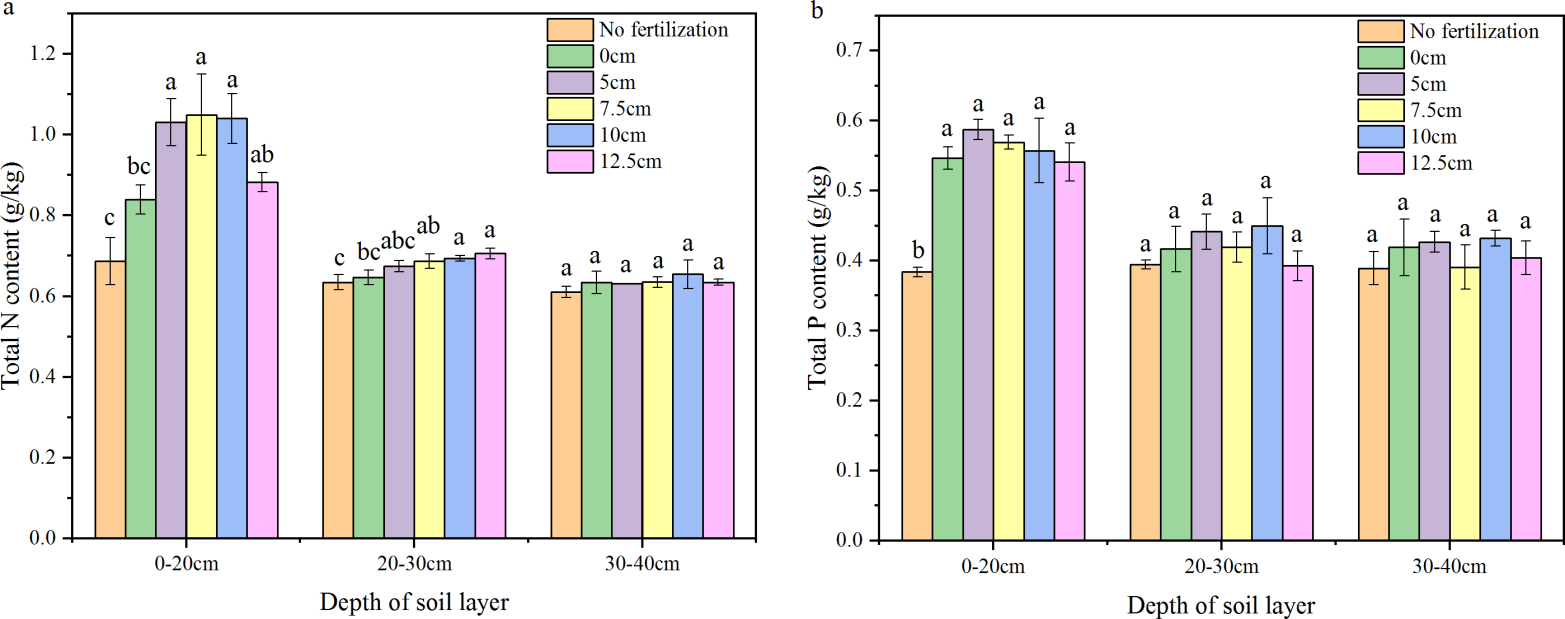
Total N and P content in the different depth of soil layer. (A) N; (B) P Different letters indicate significant differences at *P*<0.05 within the same soil layer among the treatments with different depth of fertilization.

The application of fertilizer increased the total N content in the tillage layer (020 cm) of the soil (Fig. 7). Though the total N content in the 020 cm of the soil column was 22.3% higher in surface fertilization than no fertilization, there was no significant difference between surface fertilization and no fertilization. The total N contents (020 cm) of the 5, 7.5, and 10 cm treatments were significantly higher than that of surface fertilization and no fertilization by 22.823.9% and by 50.2%-52.8%, respectively (Fig. 7; Table 1). The total N content of tillage layer (020 cm) in the 12.5 cm treatment showed no significant increase over that of surface fertilization, but was significantly higher than that of no fertilization by 28.5%. In the 2030 cm soil layer, the trend was that the total N content increased with the greater depth of fertilization. There was no significant difference among 0 cm, five cm, and no fertilization. The total N content in 7.5 cm was significant higher than no fertilization, but there was no significant increase over 0 cm. The total N content in 10 cm and 12.5 cm were significant higher than 0 cm by 7.49.2%. In the 3040 cm soil layer, there was no significant difference in total N content among the 0 cm, five cm, 7.5 cm, 10 cm, 12.5 cm, and no-fertilization treatments.

In the tillage layer of the soil column, the total P contents in fertilization treatments were significant higher than the no fertilization treatment by 40.953.0%. However, there were no significant differences among the fertilization treatments of 0, 5, 7.5, 10, and 12.5 cm. In the 2030 cm and 3040 cm soil layers, no significant difference was found in total P content among treatments of 0 cm, five cm, 7.5 cm, 10 cm, 12.5 cm, and no fertilization.

## Discussion

There is still great pressure to meet the food demands on an expanding population. The application of fertilizers, especially nitrogen and phosphorus which have great promotion to grain yield of, remains essential for food security. However, the loss of N and P from agroecosystems results in environmental problems and threatens the sustainability of agricultural practices (Nasar et al., 2020). This concern has been considered one of the major challenges to feed the world in 2050 (Ehrlich & Harte, 2015; Mueller et al., 2012). So far, the reduction of N and P losses has become an important scientific issue (Zhu & Chen, 2002; Gu et al., 2015), and a lot of research has been on the reasonable amount of fertilizer and environmentally friendly fertilizers (Fan et al., 2007; Cai et al., 2016; Chalk et al., 2015; Chen et al., 2018; Shi et al., 2012; Ahmed et al., 2014). In this paper, another approach which is simple and easy to mechanize was introduced. Deep fertilization has significantly decreased the N and P losses by NH_3_ volatilization and runoff compared with surface fertilization. With greater depth of fertilization, NH_3_ volatilization was significantly decreased, and there was no significant difference among the 10 cm, 12.5 cm, and no fertilization treatments (Fig. 2). This means that there was little N loss by NH_3_ volatilization when the fertilizer was placed below 10 cm. A similar trend was observed on the total N concentration of surface water (Figs. 3A, 4). The N leakage was increased by fertilization, but no significant difference was found among the surface fertilization and deep fertilization (Figs. 3B, 4), and the N content in the soil layer of 3040 cm also showed no significant difference (Fig. 7A), indicating that deep fertilization (512.5 cm) did not promote N loss by leaching at the depth of 40 cm compared with surface fertilization. However, there was a trend that the N content in the soil layer of 2030 cm was increased slightly with the deepening of fertilization depth (Fig. 7A). It signified that N leakage from layers of 020 cm to 2030 cm was slightly increased by greater fertilization depth. Due to the reduced loss of N, the N content in the tillage layer (020 cm) of the deep fertilization treatments was increased by 5.125.0% compared with surface fertilization treatment (Table 1). Phosphorus is more easily absorbed and held by soil compared with N, which led to better results on the reducing loss-P by deep fertilization. Unlike N, P leakage was not increased by the application of fertilizer, and there was no significant difference between each depth of fertilization and no fertilization (Figs. 5B, 6). Previous studies showed that there was a change point of soil Olsen-P, above which the potential risks of P loss by leaching increased rapidly (Hua et al., 2017; Kleinman et al., 2007). Deep fertilization was an effective way to reduce P-loss and increase P content in tillage layer soils (Table 1).

In this study, deep fertilization was confirmed to be much less harmful to the environment compared with surface fertilization (the control effect of N and P loss were calculated in Table 1). Because of the less loss of N and P, the application of fertilizer would be reduced, which may further efforts to lessen non-point pollution of N and P. Furthermore, the fertilizers used in this study were general chemical fertilizers. We believe that there would be better effect on the reducing loss of N and P using environmentally friendly fertilizers with deep fertilization. Hence, this fertilization approach has promise for cleaner agricultural production. Nevertheless, the potential popularization of deep fertilization depends on the type of machinery. Whether the fertilizer could be applied to the designated location smoothly and then covered by soil completely by the machine would be the key to success of deep fertilization. At present, the emergence of the machines used for transplanting rice seedlings with synchronous fertilization makes deep fertilization possible (Zhong et al., 2019). However, the depth of this fertilization was only five cm; in this paper, the effect of other (deeper) depths of fertilization on the N and P losses were introduced, providing some guidance for the optimal depth of fertilization.

## Conclusions

The soil column test showed that increasing the depth of fertilization within the range of 012.5 cm had little impact on the N and P losses by leaching, but significantly inhibited N losses by NH_3_ volatilization and runoff. There were significant reductions in P losses by runoff, which resulted in increased N and P content in tillage layer of the soils compared with surface fertilization. There was also no significant difference between 10 cm and 12.5 cm fertilizer depths on N and P losses by NH_3_ volatilization and runoff. Thus, Fertilization at 10 cm depth was recommend in the red paddy soil of south China.

##  Supplemental Information

10.7717/peerj.11347/supp-1 Supplemental Information 1 Raw Data

## Additional Information and Declarations

### Data Availability

The following information was supplied regarding data availability: The raw measurements are available in the Supplementary Files.
